# Role of CK7 Immunoreactivity in Predicting Progression of the Cervical Intraepithelial Neoplasia: A Retrospective Cross-sectional Study

**DOI:** 10.30699/IJP.2024.2019662.3239

**Published:** 2024-10-02

**Authors:** Soheila Sarmadi, Fatemeh Nili, Kiana Anousha

**Affiliations:** 1 *Department of Pathology, Yas Hospital Complex, Tehran University of Medical Sciences, Tehran, Iran*; 2 *Department of Pathology, Imam Khomeini Hospital Complex, Tehran University of Medical Sciences, Tehran, Ira* *n*

**Keywords:** Cervical neoplasia, CIN progression, CK7, HSIL, LSIL, Squamocolumnar junction marker

## Abstract

**Background & Objective:**

Cervical intraepithelial squamous lesions (SIL) are the first step toward developing most cervical cancers. Moreover, most of the previous studies focusing on a reliable predictive factor of cervical cancer progression have been reported as inconclusive. Recently, immunophenotyping of squamocolumnar junction cells with CK7 has been introduced as a novel SIL detection and risk evaluation method. Therefore, we aimed to investigate the value of CK7 positivity and its pattern in predicting the progression of LSIL to high-grade counterparts.

**Methods:**

This retrospective study was conducted in Yas Complex Hospital from 2016 to 2018 among patients of the clinic of colonoscopy. Patients with low-grade lesions were included in one group, and patients with high-grade lesions were considered as a control group. The immunoreactivity of CK7 by immunohistochemical staining pattern was interpreted in both groups.

**Results:**

CK7 immunoreactivity was negative in 29.5% of the precancerous lesions and positive in 70.5%. The relationship between grade and CK7 staining was significant (*P* = 0.040). A significant relationship between the progression status of the disease and CK7 staining was obtained (*P*=0.001). CK7 staining showed a sensitivity of 82.35%, specificity of 81.82%, positive predictive value of 87.5%, negative predictive value of 75%, and diagnostic accuracy of 82.14% in predicting the progression of the SILs.

**Conclusion:**

Our data and previous studies support the correlation of CK7 expression with a higher chance of progression of low-grade intraepithelial lesions.

## Introduction

Cervical squamous intraepithelial lesions (SIL) or cervical intraepithelial neoplasia (CIN) are a family of precancerous neoplasia highly linked to infection caused by the human papillomavirus (HPV) (1). They are categorized in a spectrum of low-grade to high-grade lesions depending on the persistence of HPV infection and involvement with high-risk strains (2, 3). 

CIN and SIL correspond to a spectrum of lesions with different epithelial maturation. Low-grade intraepithelial lesion (CIN1) is characterized by the presence of so-called koilocytic changes, including hyperkeratosis, dyskeratosis, multinucleation, and an intra-cytoplasmic perinuclear halo with an enlarged, irregular, and hyperchromatic nucleus (4). On the other hand, high-grade squamous intraepithelial lesions (HSIL) consist of CIN 2 and CIN 3. They are characterized by moderate to severe dysplasia with less mature cells and a higher mitotic rate as well as nuclear abnormalities (5, 6). 

Approximately 90% of CINs will regress spontaneously within 12-24 months; however, there is no consensus regarding the distinguishing factors between lesions that will regress and those that will not (7). On the other hand, nearly 14% of CIN1 lesions may progress to lesions of higher grade during follow-up (8, 9). 

Given the heterogeneity in the regression rate and the importance of conservative therapy in fertile women, establishing a reliable predictive factor for the progression of these lesions is essential (10). Neither clinical findings nor histopathologic evaluation of H&E-stained slides are practical in that regard (11). The biomarker P16 is widely used as a surrogate marker associated with high-risk HPV infection (12). However, it is recommended to be used only in equivocal cases where HSIL is in the differential diagnosis with a non-neoplastic lesion (6, 13, 14). The prognostic value of p16 in either LSIL or HSILs is also controversial (15). Rather recently, the squamocolumnar junction (SCJ) has been debated as an anatomical landmark with a discrete population of cells linked to most cervical neoplasia (16). Therefore, distinctive immunophenotyping of SCJ cells has been introduced as a novel method for CIN risk evaluation (17). One of the most debated markers is CK7, which stained SCJ cells specifically and is also positive in most CINs and carcinomas (8, 18). The association of CK7 expression with HPV16 viral oncoprotein E7 and its loss after ablative therapy suggest a possible role of CK7-positive cells in HPV oncogenesis and HPV-related cancer development (1, 19).

Considering the rapid rise of HPV infection in developing countries, we aimed to investigate the value of CK7 positivity and its pattern in predicting the progression of LSIL to high-grade counterparts. Therefore, a triage method for patients with LSIL may be established, and patients with a higher risk of progression would be monitored more closely. The finding of such a study may result in the prevention of over-treatment in low-risk patients and early detection of adverse lesions in high-risk patients.

## Material and Methods

**Fig. 1:**
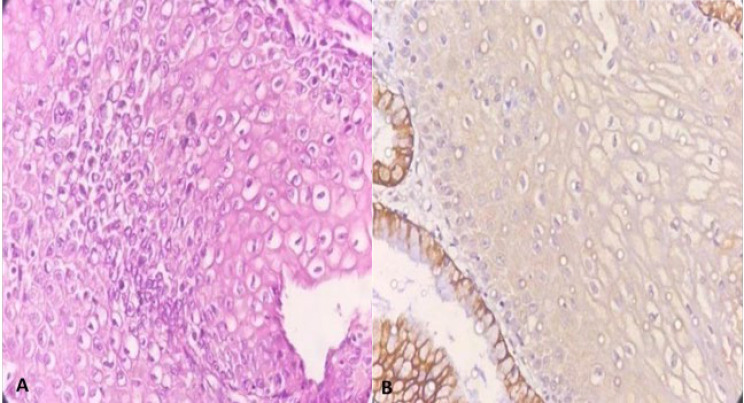
A) Normal squamocolumnar junction epithelium with B) CK7 positive staining in the endocervical cells and negative staining in the squamous epithelium (200X)

**Fig. 2:**
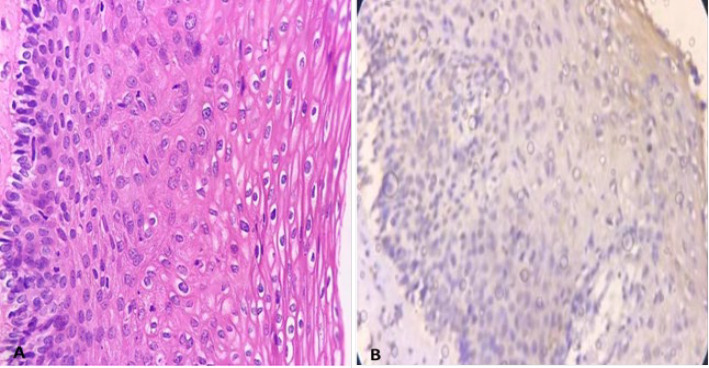
A) Low-grade squamous intraepithelial lesion (CIN1), B) Patchy weak staining for CK7 (200X)

**Fig. 3:**
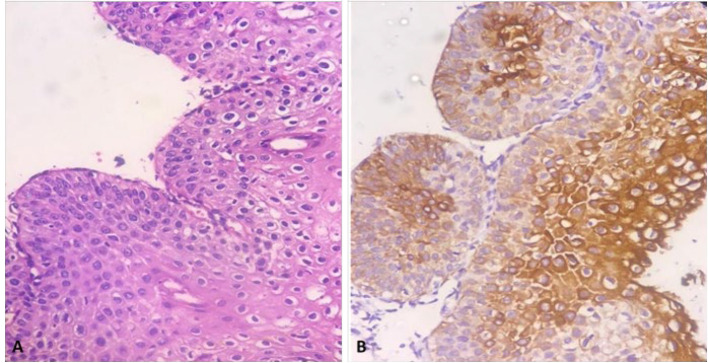
A) High grade squamous intraepithelial lesion (CIN2), B) Positive CK7 staining (200X)

**Fig. 4:**
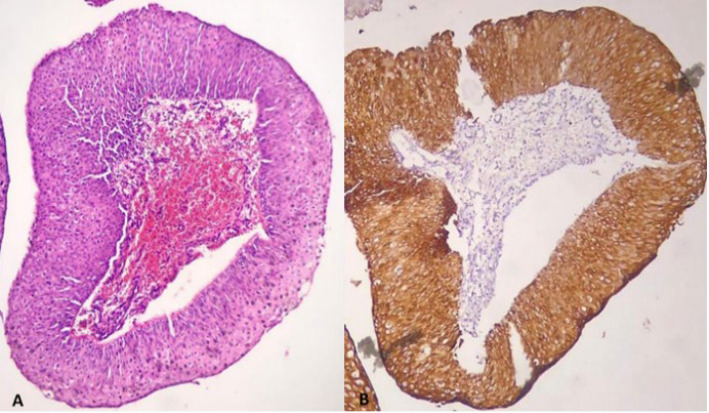
A) High-grade squamous intraepithelial lesion (CIN3), B) CK7 positivity in the full thickness of the epithelium (100X)

This retrospective cross-sectional study was conducted by reviewing the pathology files of the patients referred to the colposcopy clinic of Yas Complex Hospital from 2016 to 2018. All patients with a diagnosis of LSIL or higher with at least two years of follow-up were included. Paraffin blocks of samples with low quality and those with a small amount of residual tissue were excluded along with those patients with incomplete documented files. Ultimately F 44 patients were selected and were divided into two groups according to risk assessment. The first group, considered as low risk consisted of 23 patients with diagnosis of CIN1, CIN 1-2, and CIN 2. Patients with higher risk, including 21 patients with CIN2-3 and CIN3, were considered as the control group. In addition, clinical data, including age and progression status (progressing, regressing, or stable states of the disease), were collected by reviewing the documented files or repeated colposcopy and/or HPV testing in 2 years, respectively. H&E-stained slides were reviewed to confirm the diagnosis and selection of appropriate blocks for IHC studies. In addition, other pathological findings such as the presence or absence of cervical squamous metaplasia, atypical mitosis, presence of severe inflammation, and involvement of endocervical glands (crypts) with CIN lesions were recorded to evaluate their association with CK7 staining. The IHC staining pattern was interpreted using the model proposed in the previous studies by Herfs *et al.*, Paquete *et al.,* and finally, more fully by Mills *et al.* (13, 20, 21). The pattern of expression was categorized into four groups as follows based on the intensity and extent of staining within the lesional tissue: negative, patchy, gradient & full thickness. The absolute absence of immunoreactivity or very weak and small focal immunoreactivity of individual cells was considered negative (Figure 1). If the staining was observed in isolated clusters of 5 to 6 cells without either the reactivity of the entire epithelial thickness or decreased intensity of the staining from surface to depth, it was considered patchy (Figure 2). If at least 5 to 6 contiguous cells in a row show positive staining with a decrease in intensity or complete loss of staining from the surface to the depth of the epithelium, the pattern is called gradient (Figure 3). Finally, if at least 5 or 6 adjacent horizontal cells show immunoreactivity without decrement of intensity from surface to depth, we called it full thickness without requiring immunoreactivity of every single basal or parabasal cell (Figure 4).

## Results

The mean age in the first group was 37.5 years, ranging from 24 to 63 years old. Precancerous lesions were low-grade in 52.3% (23 of 44 cases) and high-grade in 47.7% (21 of 44 cases). CK7 immunoreactivity was negative in 29.5% (13 of 44 cases) of precancerous lesions and positive in 70.5% (31 out of 44). In more detail, CK immunostaining was negative in 22.7% (10 out of 44 cases), patchy in 6.8% (3 out of 44 cases), gradation in 43.2% (19 out of 44 cases), and full thickness in 27.3% (12 out of 44 cases). Improvement and regression were seen in 39.3% of patients. Atypical mitosis was observed in 27.2% (12 out of 44) of the patients. HPV genotyping showed high-risk genotypes in nine out of 10 patients who had undergone HPV typing and whose information was on the file. A summary of evaluated data is collected in Table 1. More than 85% of the patients with lower-grade lesions did not have severe inflammation. No correlation was found between the age of the patients and their immunoreactivity pattern. The relationship between grade and CK7 staining was significant (*P*=0.040). In low-grade lesions, CK7 staining was negative at 34.8% (eight out of 23) of the patients, and gradient staining was in 47.8% (11 out of 23). The frequency of gradient and full-thickness staining was higher in high-grade lesions. The frequency of gradient and full-thickness staining patterns in grade 2-3 lesions were 40% (two out of five) and 60% (three out of five), respectively, and the frequency of gradient and full-thickness staining patterns in grade 3 lesions were 36.4% (4 out of 11) and 54.5% (6 out of 11), respectively (Table 2).

**Table 1:** Summary of the studied variables

N (%)	Variables
Age	
16(57.1%)	37≥
12(42.9%)	37<
Grade of precancerous lesion	
23(52.3%)	Low-grade (CIN 1, CIN1-2, CIN2(
21(47.7%)	High-grade (CIN2-3, CIN3(
HPV genotyping	
9	High-risk
1	Low-risk
Follow-up results	
11(39.3%)	Regression
17(60.7%)	Persistent/ progression to CIN 2+ or conization
12(30.8%)	Atypical mitosis
CK 7 reactivity	
Negative	
10(22.7%)	Negative
3(6.8%)	Patchy
Positive	
19(43.2%)	Gradient
11(39.3%)	Full-thickness

**Table 2:** CK7 immunoreactivity in each grade

**Grade**	**CK7 immunoreactivity**			
	Negative	Patchy	Gradient	Full thickness
Low-grade	1 (4.3%)	11 (47.8%)	3 (13%)	8 (34.8%)
High-grade	11 (52.4%)	8 (38.1%)	0 (0)	2 (9.5%)

The relationship between disease status (regression, progression, persistence) and CK7 staining was statistically significant (*P*=0.001). In patients with negative staining, 72.8% showed disease improvement/regression, and 64.7% of patients with gradient staining demonstrated disease progression or persistence, and the difference was significantly higher (*P*=0.001). CK7 negative lesions were more likely to regress than CK7 positive lesions (*P*=0.001). In 12 patients with negative and patchy staining patterns, only three patients (25%) had persistent disease, and the lesions underwent spontaneous regression in the remaining nine patients (75%). In either low-grade or high-grade lesions with gradient and full-thickness CK7 staining pattern, two out of 16 patients (12.5%), showed improvement, and in the remaining 14 cases (87.5%), one case progressed to CIN 2, and the rest had persistent lesions (Table 3).

The relationship between atypical mitosis and CK7 staining was significant (*P*=0.01). Gradient and full-thickness staining patterns were seen in 50% and 41.7% of the lesions with atypical mitosis, respectively. Negative and gradient patterns were seen in 27.3% and 45.5% of the lesions with normal mitosis. Among patients in the study group, ten patients underwent HPV genotyping, in which the relationship between HPV genotype and CK7 staining was not significant (*P*=0.1). No significant relationship was noted between severe inflammation or endocervical crypt involvement with CIN and CK7 staining pattern (*P*=0.737).

CK7 staining showed a sensitivity of 82.35%, specificity of 81.82%, positive predictive value of 87.5%, negative predictive value of 75%, and diagnostic accuracy of 82.14% in predicting the progression of CINs.

**Table 3:** Frequency of different diagnoses in AGC subgroups (NOS and FN) on the first follow-up.

CK 7 immunoreactivity		Regression/Progression rate
Positive (Full thickness, gradient)	Negative (Negative, patchy)	
2 (12.5%)	9 (75%)	Regressed
14 (87.5%)	3 (25%)	Persistent, progressed, conization

## Discussion

Cervical cancer is a major public health issue in developing countries, causing over 85% of cancer mortality among women. The footstep of persistent high-grade HPV infection and its correlated precancerous lesions have been detected in nearly all cervical neoplasia (22).

Although there is no age limitation for HPV infection in sexually active women, the highest incidence is seen in women 20 to 24 years of age. However, the probability of disease clearance is higher in these young women compared to the women over age 30 due to intact immune response and rapid turnover of cervical cells (23). In our study, patients' age ranges between 24 to 65 years old, with a mean age of 37.5 years.

Precise diagnosis and classification of CINs during histopathological evaluation are essential due to different therapeutic approaches. At first glance, LSIL may appear a rather unchallenging diagnosis, but the risk of an HSIL outcome may vary between 0.5% and 13% (24, 25). In addition, inter-observer reproducibility in the diagnosis of CIN 2 is rather low, resulting in an increasing risk of over or undertreatment (26). Therefore, in concordance with the aim of this study, seeking a reliable factor for distinguishing CINs with a higher risk of progression has been established for a while. 

Moreover, it should be stated that although HPV can infect anywhere in the lower genital tract, the cervical SCJ is considered the most significant anatomical site since it is known to be the origin of most invasive cervical cancers and their precursor lesions (2). It is now proposed that cervical SCJ comprises a distinctive population of cells with a possible embryonic origin that is more prone to hosting high-grade HPV subtypes and more occurrence for neoplastic transformation (8, 11). Interestingly, SCJ markers such as CK7, AGR2, CD63, MMP7, and GDA are also invariably positive in high-grade CINs but can show either positive or negative reactions in CIN1 (27, 28). Thus, considering the topographical preference of high-grade cervical neoplasia in the SCJ and mutual immunophenotyping of neoplastic cells with SCJ cells, these markers are proposed to evaluate the risk of CIN1 progression (29, 30). 

Herfs *et al.* were pioneers in exploring the importance of CK7 expression in the triage of low-grade CINs. In their study, about 24% of low-grade CINs with positivity for SCJ markers showed progression to high-grade lesions. In contrast, all lesions with negative expression of SCJ markers remained indolent. It should be noted that they defined positive reaction as “strong diffuse cytoplasmic immunoreactivity of the entire cervical squamous preneoplastic epithelium" (16). Paquette *et al.* attempted to divide positive reactivity for CK7 into patchy, gradient, and full-thickness patterns. This subclassification led to high inter-observer variability in determining the positive reactive pattern. In addition, negative results were also complicated by background staining of immature metaplastic cells. Regardless of all the limitations, the full-thickness staining pattern was the most effective predictor of CIN progression. However, both full-thickness and gradient staining patterns were associated with doubling the risk of high-grade lesions in future biopsies. Yet, the predictive value of negative and patchy staining patterns was lower than what was reported in the study of Herfs *et al.*, as 7.4% of CK7 negative cases still progressed to CIN 2. Therefore, Paqutte believed CK7 would be more accurate in forecasting CIN 3 progression, mainly when combined with P16 and HPV genotyping (20). In our study, negative and patchy staining patterns were observed in nearly 29% of the low-grade CINs. About 71% of low-grade CINs show gradient or full-thickness reaction with CK7. The most common positive and negative staining patterns in LSIL were gradient and negative, respectively. Positive expression of CK7 was detected in about 94% of our control group, including patients with CIN2-3 and CIN3. The full-thickness reaction was observed in more than half of the cases with CIN 3, and 60 % of the cases with CIN 2-3 or CIN 3. However, CIN 2 lesions showed a variety of negative, gradient, and full-thickness patterns. The staining pattern observed in our study is similar to what was reported in previous studies. For example, in the study by Lee *et al.*, patchy and diffuse staining patterns were observed in 40% and 52% of the lesions with CIN3, respectively (27). Youssef *et al.* stated the full-thickness pattern as the dominating pattern of CK7 expression in CIN 3; however, lesions with a diagnosis of CIN 2 either showed a variety of heterogeneous patterns or lack of CK7 expression (31). Unlike previous studies, one of our samples with CIN 3 showed a negative reaction, which could be due to an inappropriate fixation process, resulting in a false negative result. However, it should be noted that, unlike previous studies, we utilized the "ready to use" IHC kit with different clones and validation methods. 

The relationship between CIN grading and CK7 staining pattern was significant (*P*=0.04) in this study. This correlation would be even stronger if the samples were grouped as high-grade lesions (HSIL) and low-grade lesions (LSIL) (*P*=00.1). Furthermore, we noticed that the intensity of staining in the high-grade lesions is higher than in the adjacent mucosa, which suggests CK7's association with adverse lesions. Our observation showed a higher chance of spontaneous regression in the lesions with a negative CK7 expression. However, the regression rate differed depending on the staining pattern, accounting for about 75% in CK7-negative lesions, including negative or patchy staining patterns. Lesions with negative CK7 staining progressed, persisted, or underwent conization in about 6% of the cases with negative and 12% of the cases with patchy staining patterns. According to the literature, the progression rate in these situations is about 4-14% (10, 32). This aligns with our study, as about 11% of lesions with negative staining patterns did not regress. However, the regression rate was about 33% in the patients with patchy staining patterns, which is a lot lower than the reported regression rate. This could be due to our limited sample size or since the biopsy was not simply representative of the main lesion. 

Low-grade lesions with gradient or full-thickness patterns regressed in nearly 12% of the cases. The other 87% of the cases either progressed, persisted, or underwent conization with a *P*=0.001, meaningfully higher than what was reported in previous studies with a *P*=0.05 (21, 33). Besides our limited sample size, the limited follow-up period, and usage of the "ready to use" IHC kit, this difference may result from the fact that we included patients with persistent lesions, those who underwent cervical conization in the 2 years, and those with progressed lesions. In contrast, most previous studies have followed the patients until progression to CIN3. 

We demonstrated that CK7 positive staining is associated with a sensitivity of 82.35%, specificity of 81.82%, positive predictive value of 87.5%, negative predictive value of 75%, and diagnostic accuracy of 82.14% to predict the progression rate of CIN. Despite a relatively good sensitivity, specificity, and PPV, the negative predictive value is relatively low. This may be due to different regression rates in different staining patterns, wrong interpretation of borderline patterns, or ablative therapy before the progression of lesions to CIN3.

Another interesting point in our study is the meaningful correlation of atypical mitosis in low-grade lesions and CK7 expression with a *P*=0.01. These lesions showed a gradient or full-thickness staining pattern. This is in concordance with the relation of CK7 positivity to HPV oncogenesis. 

Studies on the correlation between CK7 expression and age, severe inflammation, and endocervical glands' (crypt) involvement are extremely limited. No significant correlation was found in our study either. Moreover, a possible inverse relationship between HPV-L1 capsid protein expression and CK7 expression was stated in previous studies. Unfortunately, HPV genotyping was performed on a limited number of our cases due to the high cost. Hence, the absence of this association in our study is inconclusive. 

Further studies with a larger population, comparing CK7 with p16 and a more extended follow-up period, are required to illuminate other aspects of this topic.

## Conclusion

Our data and previous studies support the correlation of CK7 expression with a higher chance of CIN1 progression. While there are many limitations in interpretation of the staining patterns, positive reactivity can aid in predicting the outcome. Therefore, CK7 can assist in the triage of patients with CIN1.
